# The cup fungus *Pestalopezia brunneopruinosa* is *Pestalotiopsis gibbosa* and belongs to Sordariomycetes

**DOI:** 10.1371/journal.pone.0197025

**Published:** 2018-06-27

**Authors:** Kyoko Watanabe, Shunsuke Nozawa, Tom Hsiang, Brenda Callan

**Affiliations:** 1 Graduate School of Agriculture, Tamagawa University, Machida, Tokyo, Japan; 2 Environmental Sciences, University of Guelph, Guelph, Ontario, Canada; 3 Pacific Forestry Centre, Natural Resources Canada, Victoria, British Columbia, Canada; Universita degli Studi di Pisa, ITALY

## Abstract

*Pestalopezia brunneopruinosa*, the type species of *Pestalopezia* in Leotiomycetes, produces typical cup-shaped ascomata. Because its asexual morph has conidia comprised of five cells including apical and basal appendages and three pigmented median cells, it was first described as *Pestalotia gibbosa*, which belongs to Sordariomycetes. This contradiction has not been resolved due to the difficulty in isolating this fungus in culture. In this study, we isolated separate strains from the sexual morph and the asexual morph for molecular analysis. Phylogenetic trees of Sporocadaceae based on internal transcribed spacer, partial *β-tubulin*, and partial translation elongation factor 1-alpha sequence datasets revealed that both strains fall into the same taxon, in a clade in *Pestalotiopsis* sensu stricto alongside *P*. *gaultheriae* and *P*. *spathulata*. We provide the first evidence that fungi producing cup-shaped ascomata in *Pestalotiopsis* belong to Sordariomycetes, and we have proposed the transfer of *Pestalopezia brunneopruinosa* to *Pestalotiopsis gibbosa*.

## Introduction

*Pestalopezia brunneopruinosa* (Zeller) Seaver is a leaf spot pathogen on salal (*Gaultheria shallon* Pursh) that produces asci on an apothecium as a sexual morph [1]. The asexual morph of *Pestalopezia brunneopruinosa* resembles that of *Pestalotiopsis* sensu lato (s. lat.) and was first described independently by Harkness [2] as *Pestalotia gibbosa*. Thus, it has been suspected that *Pestalopezia brunneopruinosa* and *Pestalotia gibbosa* are the same fungus, because the two fungi have been found in close proximity on the same leaves. Bonar [3] demonstrated that cultures from germinated ascospores of *Pestalopezia brunneopruinosa* produced conidia that were the same as that of *Pestalotia gibbosa*. Seaver [4] likewise concluded that *Pestalopezia brunneopruinosa* was the sexual morph of *Pestalotia gibbosa*. However, phylogenetic analyses of both fungi to clarify their relationship has not been previously conducted.

The genus *Pestalotia* was established by De Notaris [5]. Subsequently, Steyaert [6] split the genus *Pestalotia* into *Pestalotia* sensu stricto (s. str.) (conidia composed of 6 cells), *Pestalotiopsis* (5 cells) and *Truncatella* (4 cells), although many species were still retained in *Pestalotia* s. lat. without reconsideration. Recently, *Pestalotiopsis* s. lat. was further split into three genera, *Pestalotiopsis* s. st., *Neopestalotiopsis*, and *Pseudopestalotiopsis*, based on morphology and molecular phylogeny [7]. These fungi belong to Sporocadaceae within Sordariomycetes [8]. The Harkness description of *Pestalotia gibbosa* conidia (three pigmented median cells in five-celled versicoloured conidia, with septa darker than the rest of the cell), is similar to that of *Neopestalotiopsis*. However, the current taxonomic position of *Pestalotia gibbosa* is unclear, especially since the disposition of this fungus in Sordariomycetes was made without molecular data support. The sexual morph of *Pestalotiopsis* s. lat. was determined by Barr [9] to be *Pestalosphaeria* which produces three celled-ascospores and perithecial ascocarps. Réblová et al. [10] proposed using the name *Pestalotiopsis* rather than *Pestalosphaeria* as the currently accepted name, following recent botanical code changes, but there was no mention of the name *Pestalopezia* in this argument. *Pestalopezia*, *Pestalotiopsis*, and *Pestalosphaeria*, are, however, included in a “without-prejudice list of generic names of fungi for protection under the International Code”[11].

*Pestalopezia brunneopruinosa*, the sexual morph, was classified as a member of the Leotiomycetes [12] because it produces cup-shaped ascomata. Thus, the genus names of the sexual and asexual morphs are currently forced into different taxonomic classes. Beimforde et al. [13] conducted a phylogenetic analysis by combining fossil data and molecular data (18S rDNA, 28S rDNA, RPB1, and RPB2) and showed estimated lineages of both families diverged during the Permian or Carboniferous periods and Leotiomycetes and Sordariomycetes are sister clades. Their results indicate that these families, both of which produce inoperculate asci, are closely related in the molecular phylogenetic tree. However, there is no report that fungi belonging to Sordariomycetes can produce cup-shaped ascomata. The aim of this study was to clarify the taxonomic position of *Pestalopezia brunneopruinosa* with respect to *Pestalotia gibbosa*, and to determine the name for this fungus based on the concept of one fungus, one name [14, 15].

## Materials and methods

### Sample collection and isolation

**Fig 1:**
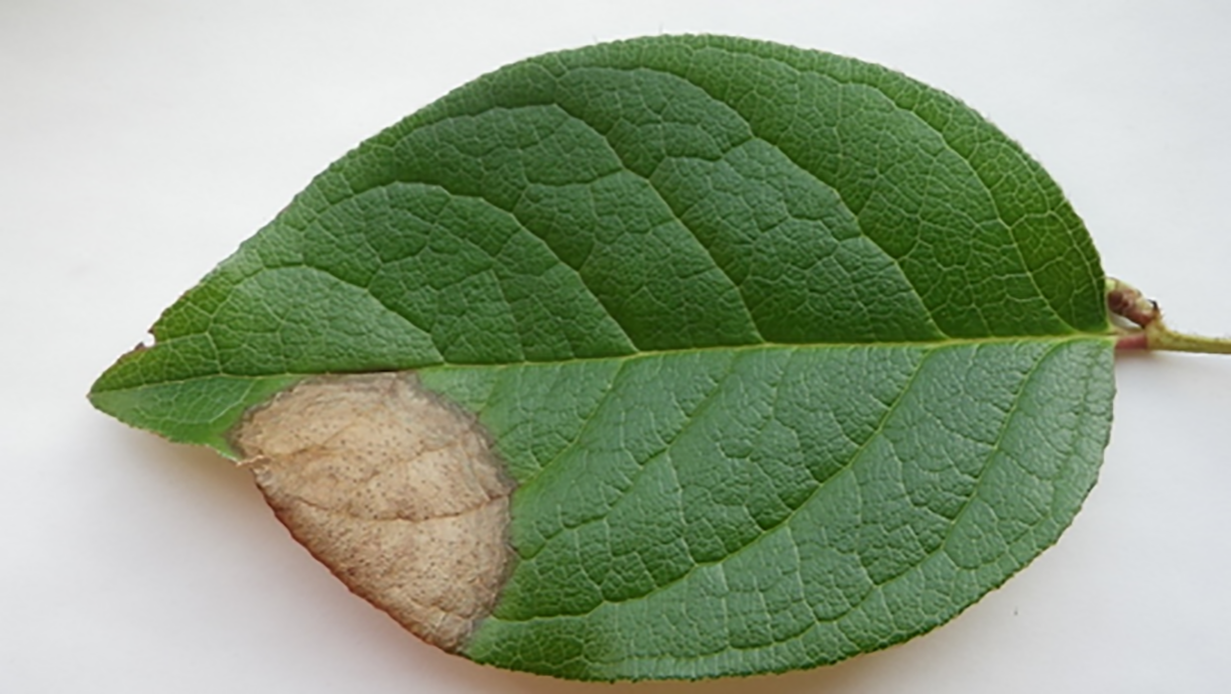
A diseased leaf of salal (*Gaultheria shallon*) from Sandcut beach trail, Vancouver Island, BC, Canada.

Diseased leaves of salal (Fig 1) were collected from Sandcut Beach trail near Shirley, Vancouver Island BC, Canada in 2013. Several isolates that originated from single conidia in acervuli were cultured from diseased leaves. Isolates were also initiated from ascospores in an ascus, but ejected ascospores failed to individually germinate. Subsequent transfers from the ascus isolate were made from single conidia. Isolates obtained from the asexual morph: NOF 3175/TAP13K_P3, and from the sexual morph: NOF 3176/TAP13K_ca_as2 were maintained on PDA (potato dextrose agar, Eiken, Tokyo, Japan) at 15°C, examined to assess taxonomic position, and deposited in The Fungus Culture Collection of the Northern Forestry Centre, Edmonton, Alberta, Canada and Tamagawa University, Machida, Tokyo, Japan. A voucher specimen containing both apothecia and acervuli was deposited in the Pacific Forestry Centre Forest Pathology Herbarium (DAVFP 29689). Information of new combination in Nomenclature was deposited in the Mycobank (http://www.mycobank.org/defaultinfo.aspx?Page=Home: MB#824630).

### DNA extraction and molecular analysis

DNA from each strain was extracted using the Qiagen DNA Mini Kit (Qiagen, Tokyo, Japan) following the manufacturer's protocol. Internal transcribed spacer (ITS), *β-tubulin*, and partial translation elongation factor 1-alpha (*tef1*) gene regions were amplified as described previously [16–19], using primers ITS1/ITS4, Bt2d/Bt2c, and pest_ef_f/EF1-1567R, respectively. These primers target regions that are approximately 550 bp, 560 bp, and 530 bp in size, respectively.

To confirm the culture was isolated from the sexual morph, DNA was extracted from a single apothecium from DAVFP 29689 by CTAB [20], and ITS was amplified using our designed primer PES3 (5’-GGCCTACCCTGTAGCGCCTT-3’) and ITS4.

Polymerase chain reaction (PCR) products were purified using ExoSAP-IT (GE Healthcare Japan, Tokyo, Japan) and sequenced using the ABI 310 DNA sequencer (ABI, Tokyo, Japan). These sequences have been deposited in the DNA Data Bank of Japan (https://www.ddbj.nig.ac.jp/index-e.html: accession numbers are shown in Table 1).

**Table 1:** Source of species for molecular analyses and the DNA database accession number.

Species	Culture No.	Location	Host	GenBank accession		
				ITS	*β-tubulin*	*tef1*
*Pestalotiopsis gibbosa* (syn. *Pestalotia gibbosa*, this study)	NOF 3175/TAP13K_P3	Canada	*Gaultheria shallon*	**LC311589**	**LC311590**	**LC311591**
*P*. *gibbosa* (syn. *Pestalopezia brunneopruinosa*, this study)	NOF 3176/TAP13K_ca_as2*	Canada	*Gaultheria shallon*	**LC311586**	**LC311587**	**LC311588**
*Pestalotiopsis adusta*	ICPM 6088	Fiji	On refrigerator door PVC gasket	JX399006	JX399037	JX399070
*P*. *anacardiacearum*	IFRDCC 2397	China	*Mangifera indica*	KC247154	KC247155	KC247156
*P*. *arceuthobii*	CBS 434.65	USA	*Arceuthobium campylopodum*	KM199341	KM199427	KM199516
*P*. *arengae*	CBS 331.92	Singapore	*Arenga undulatifolia*	KM199340	KM199426	KM199515
*P*. *australasiae*	CBS 114126	New Zealand	*Knightia* sp.	KM199297	KM199409	KM199499
*P*. *chamaeropis*	CBS 186.71	Italy	*Chamaerops humilis*	KM199326	KM199391	KM199473
*P*. *clavata*	MFLUCC 12–0268	China	*Buxus* sp.	JX398990	JX399025	JX399056
*P*. *colombiensis*	CBS 118553	Colombia	*Eucalyptus eurograndis*	KM199307	KM199421	KM199488
*P*. *diploclisiae*	CBS 115587	Hong Kong	*Diploclisia glaucescens*	KM199320	KM199419	KM199486
*P*. *ericacearum*	IFRDCC 2439	China	*Rhododendron delavayi*	KC537807	KC537821	KC537814
*P*. *furcata*	MFLUCC 12–0054	Thailand	*Camellia sinensis*	JQ683724	JQ683708	JQ683740
*P*. *gaultheriae*	IFRD 411–014	China	*Gaultheria forrestii*	KC537805	KC537819	KC537812
*P*. *grevilleae*	CBS 114127	Australia	*Grevillea* sp.	KM199300	KM199407	KM199504
*P*. *hollandica*	CBS 265.33	The Netherlands	*Sciadopitys verticillata*	KM199328	KM199388	KM199481
*P*. *humus*	CBS 336.97	Papua New Guinea	Soil	KM199317	KM199420	KM199484
*P*. *kenyana*	CBS 442.67	Kenya	*Coffea* sp.	KM199302	KM199395	KM199502
*P*. *monochaeta*	CBS 144.97	The Netherlands	*Quercus robur*	KM199327	KM199386	KM199479
*P*. *neglecta* (this study)	TAP1100*/MAFF239735	Japan	*Quercus myrsinaefolia*	AB482220	**LC311599**	**LC311600**
*P*. *novae-hollandiae*	CBS 130973	Australia	*Banksia grandis*	KM199337	KM199425	KM199511
*P*. *oryzae*	CBS 353.69	Denmark	*Oryza sativa*	KM199299	KM199398	KM199496
*P*. *papuana*	CBS 331.96	Papua New Guinea	Coastal soil	KM199321	KM199413	KM199491
*P*. *parva*	CBS 265.37	-	*Delonix regia*	KM199312	KM199404	KM199508
*P*. *pallidotheae*	MAFF 240993*	Japan	*Pieris japonica*	NR111022	LC311584	LC311585
*P*. *portugalica*	CBS 393.48	Portugal	*-*	KM199335	KM199422	KM199510
*P*. *rhododendri*	IFRDCC 2399	China	*Rhododendron sinogrande*	KC537804	KC537818	KC537811
*P*. *scoparia*	CBS 176.25	-	*Chamaecyparis* sp.	KM199330	KM199393	KM199478
*P*. *spathulata*	CBS 356.86	Chile	*Gevuina avellana*	KM199338	KM199423	KM199513
*P*. *telopeae*	CBS 114161	Australia	*Telopea* sp.	KM199296	KM199403	KM199500
*Pestalotiopsis* sp.1 (this study)	TAP0K00Kin	Japan	*Osmanthus fragrans* var. *aurantiacus*	**LC311595**	**LC311596**	**LC311597**
*Pestalotiopsis* sp.2 (this study)	TAP0E0SA*	Japan	*Camellia sasanqua*	**LC311592**	**LC311593**	**LC311594**
*Pseudopestalotiopsis cocos*	CBS 272.29	Java, Indonesia	*Cocos nucifera*	KM199378	KM199467	KM199553
*Ps*. *theae*	MFLUCC 12-0055/CPC 20281	Thailand	*Camellia sinensis*	JQ683727	JQ683711	JQ683743
*Ps*. *myanmarina*	NBRC 112264*	Myanmar	*Averrhora carambola*	LC114025	LC114045	LC114065
*Ps*. *vietnamensis*	NBRC 112252	Vietnam	*Fragaria* sp.	LC114034	LC114054	LC114074
*Neopestalotiopsis australis*	CBS 114159	Australia	*Telopea* sp.	KM199348	KM199432	KM199537
*N*. *cubana*	CBS 600.96	Cuba	Leaf litter	KM199347	KM199438	KM199521
*N*. *foedans*	CGMCC 3.9123	China	Mangrove plant	JX398987	JX399022	JX399053
*N*. *honoluluana*	CBS 114495	USA: Hawaii	*Telopea* sp.	KM199364	KM199457	KM199548
*N*. *javaensis*	CBS 257.31	Indonesia: Java	*Cocos nucifera*	KM199357	KM199437	KM199543
*N*. *natalensis*	CBS 138.41	South Africa	*Acacia mollissima*	KM199377	KM199466	KM199552
*N*. *piceana*	CBS 394.48	UK	*Picea* sp.	KM199368	KM199453	KM199527
*N*. *protearum*	CBS 114178/STE-U 1765	UK	*Picea* sp.	KM199368	KM199453	KM199527
*N*. *saprophytica*	MFLUCC 12–0282	China	*Magnolia* sp.	JX398982	JX399017	JX399048
*N*. *surinamensis*	CBS 450.74	Zimbabwe	*Protea eximia*	KM199351	KM199465	KM199518
*N*. *zimbabwana*	CBS 111495	Zimbabwe	*Leucospermum cunciforme*	JX556231	KM199456	KM199545
*Seiridium camelliae*	SD096/MFLUCC 12–0647	China	*Camellia reticulata*	JQ683725	JQ683709	JQ683741
*Seiridium* sp. 1 (this study)	TAP121	Japan	*Hamamelis japonica*	**LC311607**	**LC311608**	**LC311609**
*Seiridium* sp. 2 (this study)	TAP1041	Japan	*Chamaecyparis obtusa*	**LC311610**	**LC311611**	**LC311612**
*Seiridium* sp. 3 (this study)	TAP3355	Japan	*Tilia cordata*	**LC311601**	**LC311602**	**LC311603**
*Seiridium* sp. 4 (this study)	TAP881	Japan	*Rhododendron keiskei*	**LC311604**	**LC311605**	**LC311606**

Bold accession numbers were obtained in this study.

* indicates strain producing sexual morph.

The results of the preliminary sequence homology search using BLAST were that the two Vancouver Island salal isolates, NOF 3175/TAP13K_P3 and NOF 3176/TAP13K_ca_as2, fell into *Pestalotiopsis* s. str. Additional sequence data for phylogenetic analysis were obtained from 7 other previously unpublished strains (listed in bold in Table 1), and 43 other strains published in previous studies [7, 21]. To generate phylogenies based on ITS, *β-tubulin*, and *tef1* sequences, *Seiridium* spp., members of Amphisphaeriaceae (outgroup) and Phlogicylindriaceae, were chosen because they are phylogenetically close to Sporocadaceae. The dataset of each genomic region (ITS, *β-tubulin*, and *tef1*) was aligned using MAFFT [22]. All positions containing gaps and missing data were deleted from the analysis. The strength of internal branches from the resulting tree was tested using the bootstrap analysis [23] with 1,000 replications.

Sequence data comprising the aligned dataset were subjected to maximum-likelihood (ML), neighbor-joining (NJ) and maximum-parsimony (MP) phylogenetic analyses using MEGA software Version 7 [24]. Molecular analyses using the ML method were performed using HKY*+G+I* nucleotide substitution model for ITS, *β-tubulin*, and *tef1*. Initial trees for the heuristic searches were automatically generated by applying the NJ and BioNJ algorithms to a matrix of pairwise distances estimated using the maximum composite likelihood approach and then selecting the topology with a higher log-likelihood value. Evolutionary history was inferred using the NJ method [25]. The tree was drawn to scale with branch-length units equivalent to those of the evolutionary distances used to infer phylogeny. Evolutionary distances were computed using the Kimura 2-parameter method [26] as the number of base substitutions per site. MP trees were generated using the tree-bisection-regrafting (TBR) algorithm and search level 3, which generates initial trees by randomly adding sequences (10 replicates). Consistency, retention, homoplasy, and composition indices were calculated for parsimony-informative sites. The resulting trees were printed using TreeView v. 1.6.6 [27] and, together with the alignments, deposited as S21431 in TreeBASE (https://www.treebase.org/treebase-web/home.html).

### Morphological observations

Morphological observations were made from symptomatic salal leaves collected in 2013 (DAVFP 29689) and from a single dried herbarium specimen DAVFP 11308. The latter was collected in 1959, also from Vancouver Island, and determined as *Pestalopezia brunneopruinosa* by W. Ziller (S1 Fig). The asexual and sexual morphs were observed and measured in water using light microscopy (BX 51, Olympus Tokyo, Japan).

### Nomenclature

The electronic version of this article in Portable Document Format (PDF) in a work with an ISSN or ISBN will represent a published work according to the International Code of Nomenclature for algae, fungi, and plants, and hence the new names contained in the electronic publication of a PLOS ONE article are effectively published under that Code from the electronic edition alone, so there is no longer any need to provide printed copies.

In addition, new names contained in this work have been submitted to MycoBank from where they will be made available to the Global Names Index. The unique MycoBank number can be resolved and the associated information viewed through any standard web browser by appending the MycoBank number [urn:lsid:mycobank.org:Mycobank:824630] contained in this publication to the prefix http://www.mycobank.org/MB/. The online version of this work is archived and available from the following digital repositories:PubMed Central, LOCKSS.

## Results

### Phylogenetic analysis

**Fig 2:**
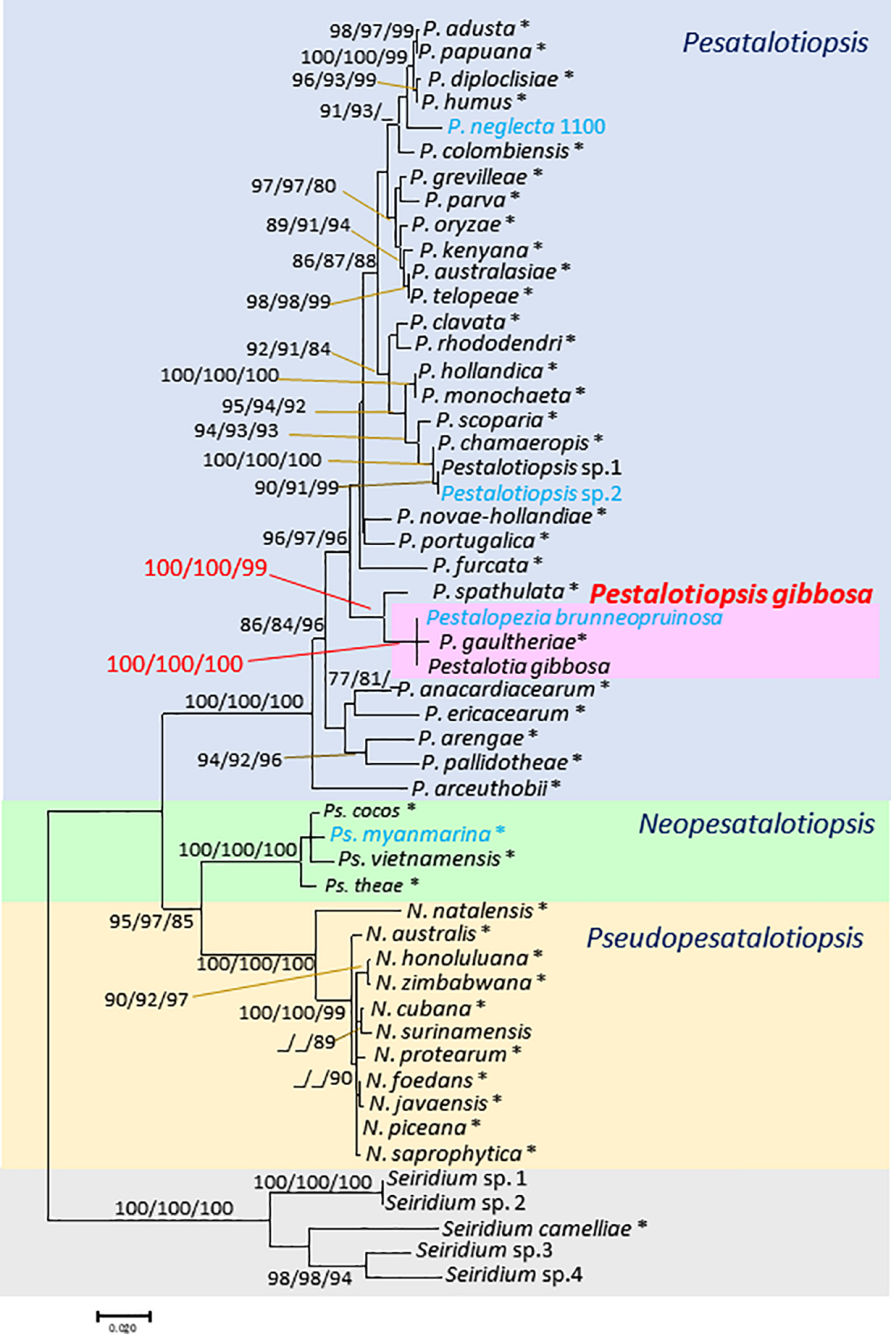
Maximum-likelihood (ML) tree with length 743 determined by analysis of the combined ITS, *β-tubulin*, and *tef1* sequence matrix. Numbers (ML/NJ/MP) and hyphens on the branches represent the bootstrap values (%) for each node, calculated from 1,000 replicates; only values > 80% are shown. NJ: neighbor-joining, MP: Maximum-parsimony. *: ex-holotype cultures. Blue texts indicate strains producing sexual morphs.

In addition to the Vancouver Island collections preliminarily identified as *Pestalotia gibbosa* (NOF 3175/TAP13K_P3, Culture from conidia) and *Pestalopezia brunneopruinos*a (NOF 3176/TAP13K_ca_as2, Culture from ascospores), a total of 52 strains, including *Pestalotiopsis* (30 strains with two obtained from sexual morphs), *Neopestalotiopsis* (11 strains), and *Pseudopestalotiopsis* (4 strains including one obtained from the sexual morph), were examined (accession numbers shown in Table 1). The sequence matrix used for phylogenetic analyses contained at least 1258 nucleotide positions for final data set from sequences 550 bp of ITS, 560 bp of *β-tubulin*, and 530 bp of *tef*1. In ML method, the highest log-likelihood was -6657.97. The optimal tree generated using the NJ method had a branch-length of 0.665. An MP tree had a length of 909, consistency index of 0.547, retention of 0.87 and composite index of 0.509. Only the ML tree (Fig 2) is shown here, because the ML, NJ, and MP methods generated similar topologies.

*Pestalotia gibbosa* and *Pestalopezia brunneopruinosa* were placed in the same clade with *Pestalotiopsis gaultheriae* (ML/NJ/MP: 100/100/100). *Pestalotiopsis spathulata* was also closely placed to *Pestalotia gibbosa* and *Pestalopezia brunneopruinosa* with highly supported bootstrap values (ML/NJ/MP: 100/100/99). Furthermore, the ITS sequence obtained from *Pestalotia gibbosa* (NOF 3175/TAP13K_P3) and *Pestalopezia brunneopruinosa* (NOF 3176/TAP13K_ca_as2) were the same as the ITS sequence obtained from DNA extracted directly from an apothecium of DAVFP 29689 (epitype specimen) (S2 Fig).

### Morphological comparisons

**Fig 3:**
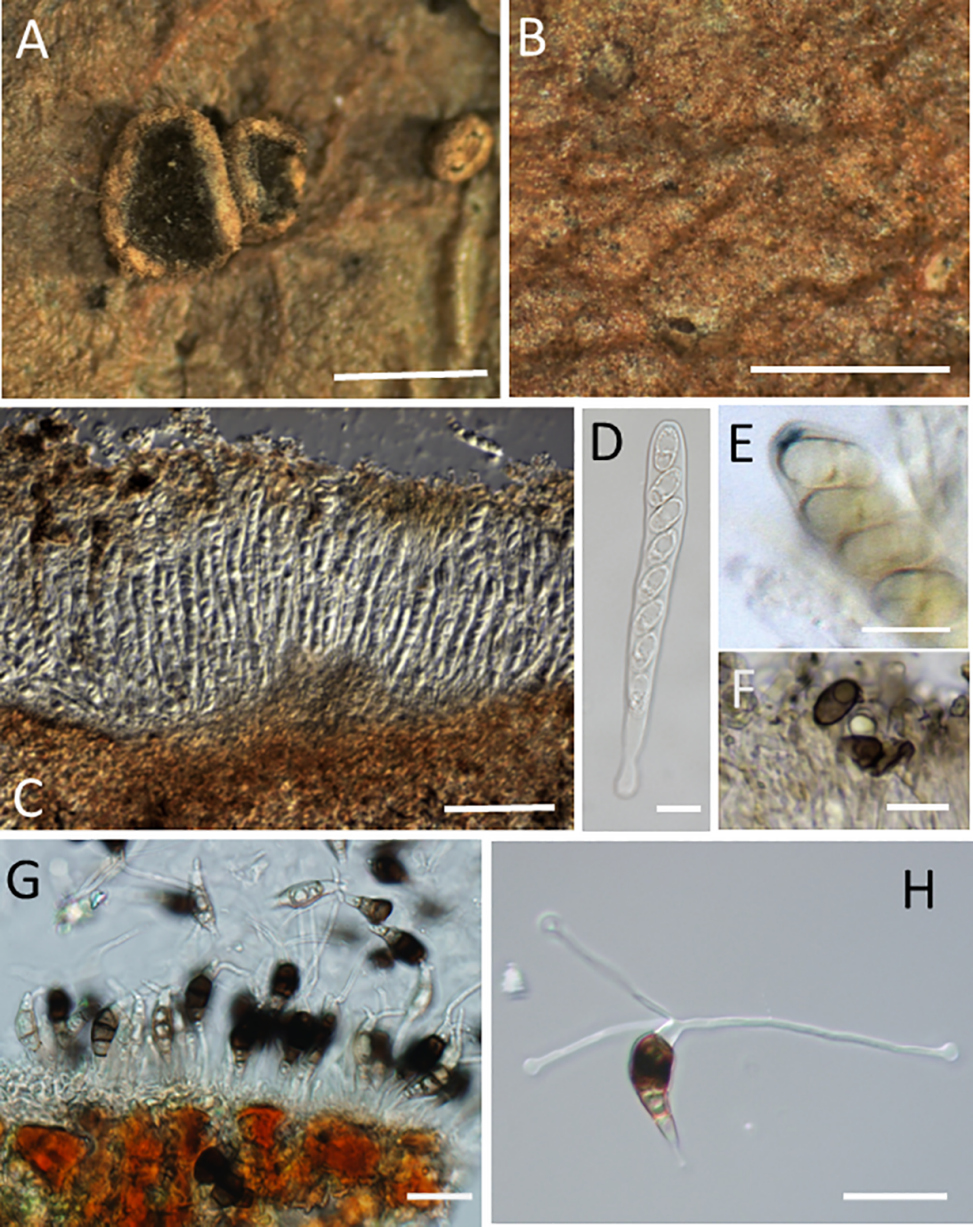
Morphological characteristics of *Pestalotiopsis gibbosa* (Epitype). (A) Apothecia (arrowheads). (B) Acervuli (arrows). (C) A vertical section of an apothecium. (D) Asci containing ascospores. (E) Apical ring of ascus tip staining blue in Melzer’s reagent. (F) Mature ascospores (G) A Section of an acervulus. (H) Conidia. Bars (A), (B) = 2 mm, (C)–(E) = 20 μm.

Our observations of the apothecia from DAVFP 11308 and 29689 are similar to those of Seaver’s description [4] of *Pestalopezia brunneopruinosa*, with few exceptions. Seaver’s ascospore measurements were slightly larger than the Vancouver Island DAVFP (VI) specimens at 7–10 x 14–20 um, plus we observed in the VI collections that mature ascospores eventually darken to brown rather than remaining hyaline (Fig 3, S3 Fig). We also observed a ring-shaped ascus apparatus in DAVFP 29689 which stained blue in Melzer’s reagent, but only in scattered mature asci. These morphological variations are relatively minor and likely reflective of the state of maturity of Seaver’s material (S1 Table). We also compared our observations and measurements of the conidial states of DAVFP 11308 and 29689 from leaves to published descriptions and specimens of conidia of *Pestalopezia brunneopruinosa*, *Pestalotiopsis gaultheriae*, and *P*. *spathulata* (Table 2). With the exception that *P*. *spathulata* has fewer and longer appendages [7], all are morphologically very similar.

**Table 2:** Morphological comparison of asexual morphs of *Pestalotiopsis gibbosa* and related species.

Species	Three median cells			Apical appendages		
	Size (length × width, μm)	Length (μm)	Colour	Number	Size (length, μm)	Tip
*Pestalopezia brunneopruinosa* ^[^^2^^]^	25–30 × 8–10.5	16–20	concolorous, olivaceous	2–4 (3)	30–60	knobbed
*Pestalopezia brunneopruinosa* (DAVFP 11308)	22.5–32 × 8–13.5	13–20	versicolorous, dark brown	1–4 (3)	20–48	knobbed
*Pestalotiopsis gibbosa* (DAVFP 29689)	24–31 × 7.5–10	15.5–22.5	versicolorous, dark brown	2–4 (3)	22–61	knobbed
*Pestalotiopsis gaultheriae* ^[^^28^^]^	23–31 × 7–9.5	–	versicolorous, dark brown	3	15–50	knobbed
*Pestalotiopsis spathulata* ^[^^7^^]^	24–32 × 7.5–9.5	13–20	versicolour	2–5	17–25	knobbed

### Taxonomy

*Pestalotiopsis gibbosa* (Harkn.) Kyoko Watan., Nozawa & B. Callan, **comb. nov.** [urn:lsid:mycobank.org:Mycobank:824630]

= *Pestalotia gibbosa* Harkn. Bull. Calif. Acad. Sci. 2: 439, 1887 MB#191515
= *Dermatea brunneopruinosa* Zeller, Mycologia 26: 291, 1934 MB#259032
= *Pestalopezia brunneopruinosa* (Zeller) Seaver, Mycologia 34: 300, 1942 MB#289174
≡*Pestalotiopsis gaultheriae* Y.M. Zhang, Maharachch. & K.D. Hyde, Sydowia 65: 121, 2013 MB#803236

### Epitype (Fig 3)

DAVFP 29689, Sandcut Beach trail, Shirley, Vancouver Island BC, Canada, 48.4173°N, 124.0185°W March 5, 2013, on leaves of *G*. *shallon* Pursh collected by B. Callan and M. Brannigan. Ex-epitype NOF 3176/TAP13K_ca_as2 was isolated from a conidium transferred from a colony originating from a single ascus.

*Ascocarp*: Apothecium developing on the upper surface of pale tan to light brown necrotic areas of attached living leaves, sessile or with short stalk approximately 0.5–2 mm in diameter, cup-shaped, with a wood brown to yellowish brown furfuraceous exterior. *Hymenium* fuscous when immature, becoming black at maturity because of the dark tips of paraphyses forming the epithelium; *asci*: 115–150 μm in length (including a short stalk) × 11–15 μm in diameter (n = 20), eight-spored, unitunicate, cylindrical, with slightly pointed apex, apical apparatus ring-shaped and staining blue in Melzer’s reagent, but only when fully mature; *ascospores*: 5–8 × 11–16 μm (n = 20), ellipsoidal to ovate, at first hyaline, becoming dark brown when mature, one-seriate; *paraphyses*: slender and clavate, light brown at their tips in Melzer’s reagent.

*Conidiomata*: Acervuli erumpent through the upper surface of the leaf epidermis, frequently in a zonate pattern in necrotic lesions. Lesions frequently coalescing, turning the leaf almost entirely brown while still attached to the stem. Conidiomata from leaves, subglobose to oval, immersed, then erumpent, black, up to 150–219 μm wide (n = 10); Conidiogenous cells directly lining the acervular wall, hyaline, cylindrical, annellidic; *Conidia*: 24–31 × 7.5–10 μm (n = 30), pyriform, curved, four-septate and slightly constricted at the septa, which are darker than the body of the cells; median three cells 15.5–22.5 μm long (n = 30) in total, pigmented; two upper pigmented cells fuscous, darker than lower pigmented cell, 15.5–22.5 μm long (n = 30); apical cell: hyaline, conical with two to four (mostly three) apical appendages arising from the apical crest. Apical appendages typically swollen at the tip, unbranched, filiform, 22–61 μm long (n = 30). Basal cell hyaline, conical, with a single, tubular, unbranched, centric appendage.

Additional specimen examined: DAVFP 11308 (S3 Fig), Cowichan Lake, Vancouver Island, BC, Canada, April 23, 1959, on leaves of *G*. *shallon* Pursh collected and determined as *Pestalopezia brunneopruinosa* by W. Ziller.

Note: The Holotype was O. S. C. Herb., 8096 in the original description of *Dermatea* by Zeller in 1934 [1].This description did not mention the color of mature ascospores. DAVFP 11308 collected by Ziller (as *Pestalopezia*) in 1959 contains mature ascomata and is in sufficiently intact state to observe brownish ascospores. However neither sample was suitable for DNA extraction, and hence we established an epitype. Since obtaining cultures that originate from single ascospores is difficult, we initiated our culture (NOF 3176/TAP13K_ca_as2) from a monoconidial isolate that was obtained from hyphae grown from ascospores of a single ascus. We were able to germinate single ascospores ejected from mature ascocarps onto agar, but the resulting germinants failed to grow beyond an initial germ tube. We designated the epitype of *Pestalotiopsis gibbosa* as DAVFP 29689. We consider *P*. *gaultheriae* Y.M. Zhang, Maharchch. & K.D. Hyde [28] to be a synonym of *P*. *gibbosa*, but the authors [28] were unable to obtain living cultures from the specimen of *P*. *gaultheriae*.

## Discussion

Our morphological observations and sequence results confirm that *Pestalopezia brunneopruinosa* and *Pestalotia gibbosa* are the same fungus. Conidia of *Pestalotia gibbosa* are strikingly similar to those of *Neopestalotiopsi*s species because the three median cells of the conidia are versicoloured, and they could be classified into the genus *Neopestalotiopsis* based on morphology. However, in this study, we demonstrate by genomic analysis that *P*. *gibbosa* should be transferred to *Pestalotiopsis* s. str., even though its sexual morph is an apothecium.

The majority of the more than 200 species associated with the well-known genus *Pestalotiopsis* s. lat. are typified by the asexual morph, while only a few (14) have known sexual states producing perithecial ascocarps typified by the genus *Pestalosphaeria* [7, 21]. Réblová et al. [10] have recommended use of *Pestalotiopsis* rather than *Pestalosphaeria*, but this recommendation did not take into consideration the potential of either *Neopestalotiosi*s or *Pseudopestalotiopsis* also having teleomorphs genetically related to *Pestalosphaeria*; and the small (three known species), obscure genus *Pestalopezia* was not mentioned at all in this recommendation. All species of *Pestalosphaeria* were considered to be linked to *Pestalotiopsis* s. str. after the three genera *Neopestalotiopsis*, *Pseudopestalotiopsis*, and *Pestalotiopsis* were separated from *Pestalotiopsis* s. lat. [7]. Silvério et al [29] in 2016 and Nozawa et al. [17] in 2017, found the sexual morphs of *Neopestalotiopsis* and *Pseudopestalotiopsis*, both in agreement with the description of *Pestalosphaeria*. Hence, they reported that *Pestalotiopsis* s. str., *Neopestalotiopsis*, and *Pseudopestalotiopsis* produce the same sexual morph. However, the relationship of these fungi to *Pestalopezia*, characterized by the production of apothecia, was not considered in these works. In this study, we obtained strains from conidia of *Pestalotia gibbosa* and from ascospores of *Pestalopezia*. In phylogenetic analyses based on ITS, *β-tubulin*, and *tef1*, both strains were placed with *Pestalotiopsis* s. str. (Fig 2) although the morphological characteristics of conidia were strikingly similar to those of conidia of *Neopestalotiopsi*s (Fig 3). Hence, the name of *Pestalotia gibbosa* should be changed to *Pestalotiopsis gibbos*a. Although *Pestalopezia* Seaver 1942 precedes *Pestalotiopsis* Steyaert 1949, we recommend using *Pestalotiopsis* s. str. as this name is more widely known and therefore likely to be better accepted. The species name *gibbosa* (1887) is older than *brunneopruinosa* (1942).With our strains, *P*. *gaultheriae* belongs to same clade with high bootstrap values (MP/ML/NJ: 100/100/100, Fig 2). *Pestalotiopsis gaultheria*e was established as a new species based on morphology and molecular data of ITS, *β-tubulin* and *tef1* sequences, which were directly obtained from the fungi on a leaf of salal. However, our sequence data demonstrated that *P*. *gaultheriae* was a synonym of *Pestalotiopsis gibbosa*. In sordariomycetes, there is no fungi producing cup–shaped ascomata. According to results of Zhuang et al [30] based on a phylogenetic tree of RNA secondary structures and on the estimated morphologies from their phylogenetic tree, ascomata having exposed hymenia are estimated as ancestral morphs. Even *Pestalotiopsis* s. lat. produces closed ascomata, and only the clade of *Pestalotiopsis gibbosa* produces open ascomata, nested among other taxa with closed ascomata. In this study, we were unable to determine whether this is the ancestral morph or a reversion morph. Our results provide the first evidence that Sordariomycetes include species that produce cup-shaped ascomata.

## Supporting information

S1 Fig Specimen of DAVFP 11308. This specimen is preserved in the Forest Pathology Herbarium at the Pacific Forestry Center, Victoria, BC, Canada.
(TIF)

S2 Fig Multiple alignment of ITS sequences among *Pestalopezia brunneopruinosa* (NOF 3176/TAP13K_ca_as2), *Pestalotiopsis gibbosa* (NOF 3175/TAP13K_P3), and extract DNA directly from an apothecium on DAVFP 29689. (TIF)

S3 Fig Morphological characteristics of *Pestalotia gibbosa* (DAVFP 11308). **(**A) Apothecia; (B) Acervuli; (C) Asci containing mature ascospores (arrow) on the layer of an apothecium; (D) Asci and ascospores (stained with iodine); (E) Conidial formation on the upper layer of an acervulus; and (F) Conidia. Bars (A), (B): 2 mm, (C): 100 μm, (D)–(F): 20 μm.
(TIF)

S1 Table Morphological comparison of sexual morph of *Pestalopezia brunneopruinosa* and related species. (DOCX)
