# Supplementary figures and images for: The cup fungus Pestalopezia brunneopruinosa is Pestalotiopsis gibbosa and belongs to Sordariomycetes
Source: PLoS One. 2018 Jun 27;13(6):e0197025. doi: 10.1371/journal.pone.0197025 (PMC6021046; doi:10.1371/journal.pone.0197025)

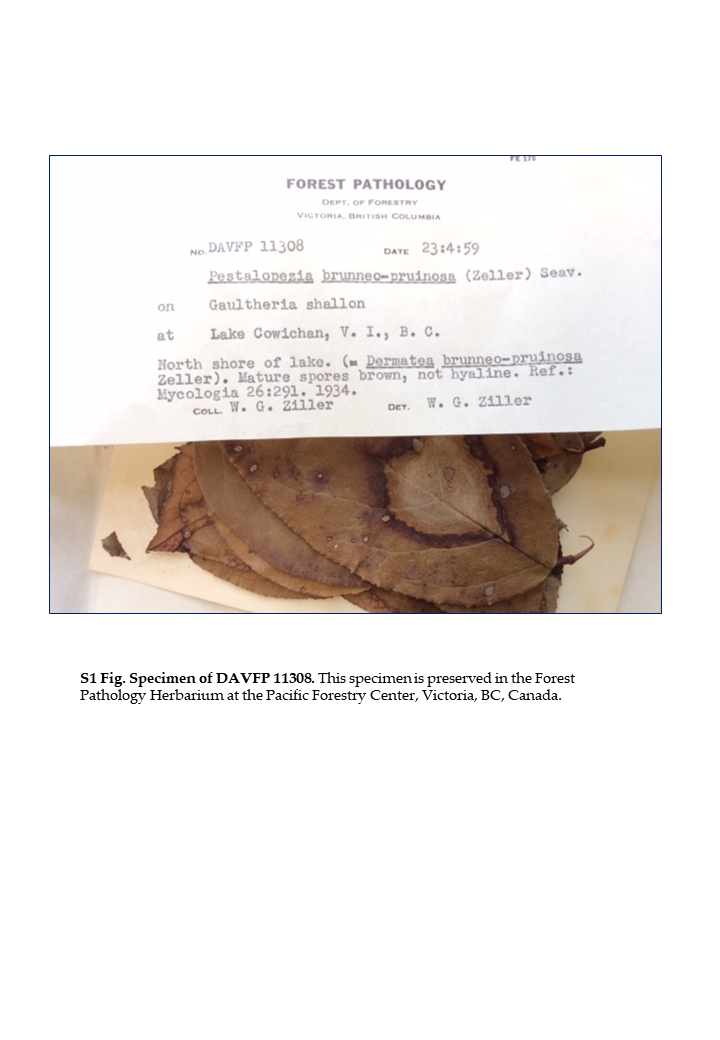

Supplement: S1 Fig — This specimen is preserved in the Forest Pathology Herbarium at the Pacific Forestry Center, Victoria, BC, Canada. (TIF) [file pone.0197025.s001.tif]

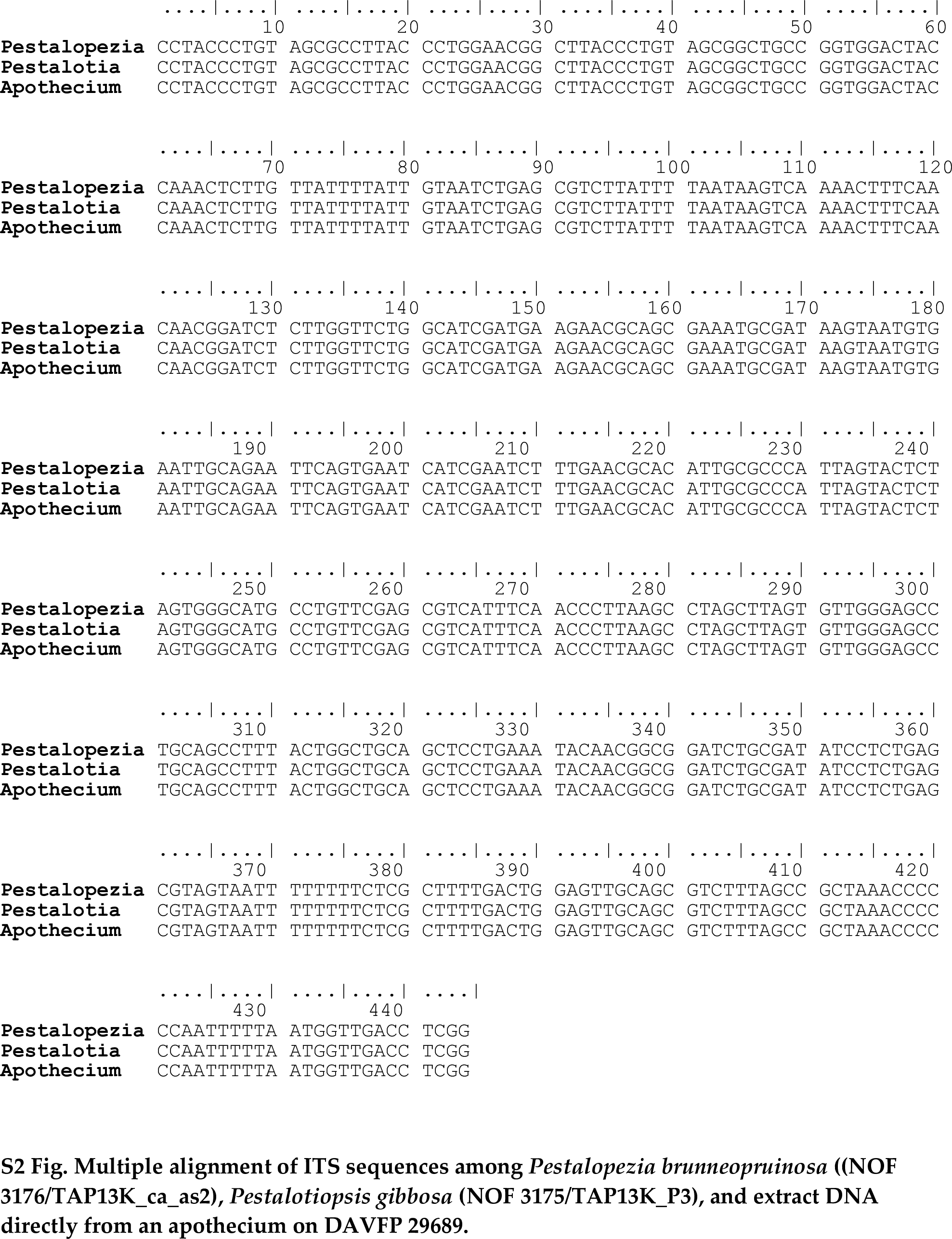

Supplement: S2 Fig — (TIF) [file pone.0197025.s002.tif]

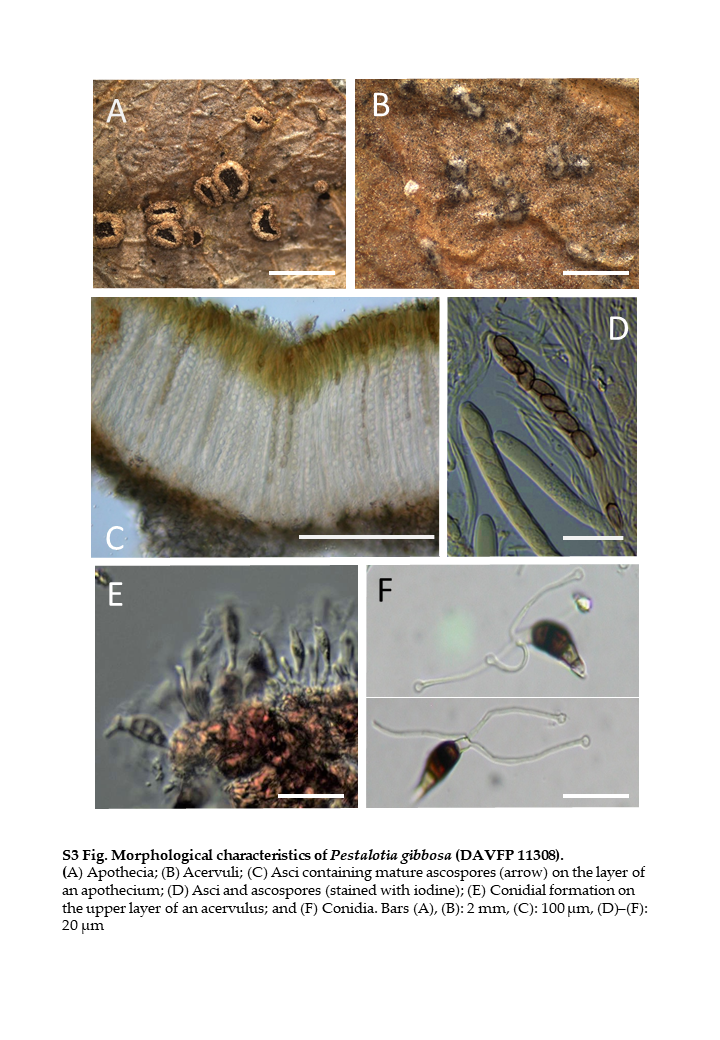

Supplement: S3 Fig — (A) Apothecia; (B) Acervuli; (C) Asci containing mature ascospores (arrow) on the layer of an apothecium; (D) Asci and ascospores (stained with iodine); (E) Conidial formation on the upper layer of an acervulus; and (F) Conidia. Bars (A), (B): 2 mm, (C): 100 μm, (D)–(F): 20 μm. (TIF) [file pone.0197025.s003.tif]
