# Supplementary material for: The cup fungus Pestalopezia brunneopruinosa is Pestalotiopsis gibbosa and belongs to Sordariomycetes
Source: PLoS One. 2018 Jun 27;13(6):e0197025. doi: 10.1371/journal.pone.0197025 (PMC6021046; doi:10.1371/journal.pone.0197025)
Supplement: S1 Table — (DOCX) [file pone.0197025.s004.docx]

**S1 Table Morphological comparisons of sexual morph of *Pestalopezia brunneopruinosa* and related species.**

| Species | Apothecia (width, mm) | Asci (length × width, µm) | Ascospores characteristics | | |
| --- | --- | --- | --- | --- | --- |
|  |  |  | Size (µm) | Colour | Shape |
| *Pestalopezia brunneopruinosa* Harkn^a^ | 0.6–2 | 120–140 × 10–14 | 7–10 × 14–20 | hyaline | ellipsoidal |
| *Pestalopezia brunneopruinosa* (DAVFP 11308) | –2 | 105–128 × 10–17 | 7–9.5 × 15–18.5 | hyaline to brown | ellipsoidal |
| *Pestalotiopsis gibbosa* (DAVFP 29689) | 0.5–2 | 115–150 × 11–15 | 5–8 × 11–16 | hyaline | ellipsoidal to ovate |
| *Pestalopezia rododendri* Seaver^[4]^ | – | 150 ×14 | 8 × 16 | hyaline | ellipsoidal to ovate |
